# Infection fatality rate and infection attack rate of COVID-19 in South American countries

**DOI:** 10.1186/s40249-022-00961-5

**Published:** 2022-04-06

**Authors:** Salihu Sabiu Musa, Amna Tariq, Liu Yuan, Wei Haozhen, Daihai He

**Affiliations:** 1 Department of Applied Mathematics, Hong Kong Polytechnic University, Hong Kong, China; 2 Department of Mathematics, Kano University of Science and Technology, Wudil, Nigeria; 3 Department of Population Health Sciences, School of Public Health, Georgia State University, Atlanta, GA USA

**Keywords:** COVID-19, Epidemic model, Infection fatality rate, Infection attack rate, Pandemic, Reproduction number

## Abstract

**Background:**

The ongoing COVID-19 pandemic hit South America badly with multiple waves. Different COVID-19 variants have been storming across the region, leading to more severe infections and deaths even in places with high vaccination coverage. This study aims to assess the spatiotemporal variability of the COVID-19 pandemic and estimate the infection fatality rate (IFR), infection attack rate (IAR) and reproduction number (${R}_{0}$) for twelve most affected South American countries.

**Methods:**

We fit a susceptible-exposed-infectious-recovered (SEIR)-based model with a time-varying transmission rate to the reported COVID-19 deaths for the twelve South American countries with the highest mortalities. Most of the epidemiological datasets analysed in this work are retrieved from the disease surveillance systems by the World Health Organization, Johns Hopkins Coronavirus Resource Center and Our World in Data. We investigate the COVID-19 mortalities in these countries, which could represent the situation  for the overall South American region. We employ COVID-19 dynamic model with-and-without vaccination considering time-varying flexible transmission rate to estimate IFR, IAR and ${R}_{0}$ of COVID-19 for the South American countries.

**Results:**

We simulate the model in each scenario under suitable parameter settings and yield biologically reasonable estimates for IFR (varies between 0.303% and 0.723%), IAR (varies between 0.03 and 0.784) and ${R}_{0}$ (varies between 0.7 and 2.5) for the 12 South American countries. We observe that the severity, dynamical patterns of deaths and time-varying transmission rates among the countries are highly heterogeneous. Further analysis of the model with the effect of vaccination highlights that increasing the vaccination rate could help suppress the pandemic in South America.

**Conclusions:**

This study reveals possible reasons for the two waves of COVID-19 outbreaks in South America. We observed reductions in the transmission rate corresponding to each wave plausibly due to improvement in nonpharmaceutical interventions measures and human protective behavioral reaction to recent deaths. Thus, strategies coupling social distancing and vaccination could substantially suppress the mortality rate of COVID-19 in South America.

**Graphical Abstract:**

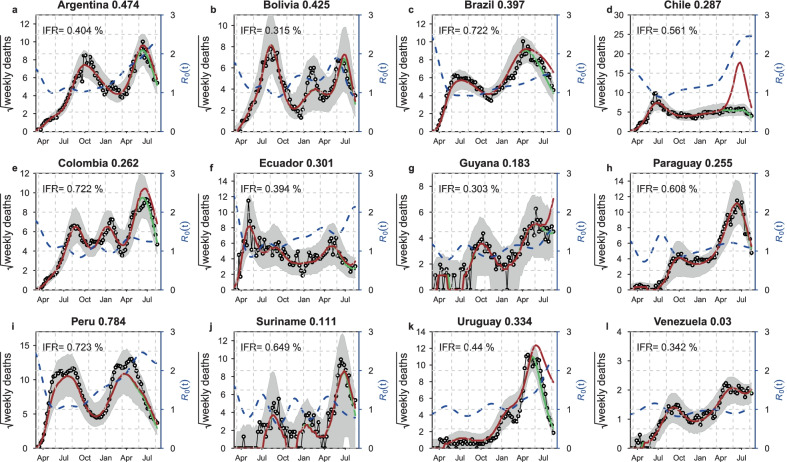

**Supplementary Information:**

The online version contains supplementary material available at 10.1186/s40249-022-00961-5.

## Background

The world has been facing devastating public health and socioeconomic growth problems in the wake of the ongoing COVID-19 pandemic [1, 2]. As of September 29, 2021, the pandemic has caused more than 230 million cases and over 4.7 million deaths across the globe [2]. The first countries/regions to be hit by the COVID-19 pandemic were China, Europe, North America, followed by the rest of the world, including South America, Africa, and the Western Pacific [3–5]. The two countries in South America with the lowest COVID-19 morbidity and mortality cases are Guyana and Suriname, which reported 792 and 893 deaths, respectively, by September 29, 2021 [6, 7]. Moreover, Brazil and Peru are two of the hardest-hit countries in terms of either total deaths or deaths *per capita* in South American region, with the second wave heavily driven by new variants, such as, P.1 variant [2, 8, 9]. By May 31, 2020, about 75.3% (4,196 of 5,570) municipalities across all five administrative regions of Brazil reported COVID-19 cases, including 206,555 (40.2%) recoveries and 29,314 (17.5%) fatalities [10]. Subsequently, by July 28, 2021, the total deaths in Brazil and Peru were 553,272 and 196,138, respectively. In particular, the Peruvian government updated its COVID-19 data and reported that 0.5% of the population had died of the disease. This caused Peru and Brazil to be the first and seventh-most COVID-19 affected countries globally in terms of deaths *per capita* rate (https://coronavirus.jhu.edu/data/mortality). Moreover, owing to the increased morbidity and mortality cases of COVID-19 in South America, the situation was especially challenging in the Amazon region, following high infection attack rates [10, 11].

The rate of COVID-19 vaccination is dramatically increasing worldwide, and South American countries are scrambling to catch up by creating openings for vaccine diplomacy to reach the target for vaccinating at least 60–70% of the population. The region represents approximately 16% and 24% of the global cases and deaths as of October 4, 2021. However, only about 7% of the worldwide vaccine doses have been administered in South America, according to the World Health Organization (WHO) report [2]. In comparison, as of October 6, 2021, at least one dose of a COVID-19 vaccine has been administered to over 46% of the world’s population. And more than 6 billion doses of vaccines have been administered so far, globally, with over 23.6 million administered each day [12], see Additional file 1: Table S1.

Although several effective vaccines are currently available [13], yet, the nonpharmaceutical interventions (NPIs) measures and other factors (such as the influence of human behavior and provision of adequate medical resources) [11, 14] continue to play significant roles in the apparent flattening of the epidemic curves and help in reducing the mortality of COVID-19 across the globe. Such as changes in human behavior, social distancing, and usage of face masks [11]. Other factors/control measures include plausible pre-existing serological cross-reactivity against SARS-CoV-2 [15], herd immunity [16], availability of medical resources [17], meteorological factors [18, 19], reduction in global transportation [20, 21], and use of effective mask [22]. These factors have resulted in highly geographical heterogeneity for COVID-19 transmission. Moreover, the spatiotemporal variability of COVID-19 pandemic has been studied across different levels through various indices [23–25]. Some countries in South America, such as Chile, have portrayed significant positive impact following implementing NPIs and other containment measures against COVID-19, including localized lockdowns, banning of large gatherings, night-time curfew, and school and country border closures [26]. These control measures helped significantly in suppressing the morbidity and mortality rates [26, 27]. The dynamic data dashboards (such as the Johns Hopkins Coronavirus Resource Center) reporting COVID-19 cases and deaths highlighted significant geographical variations in the epidemic patterns of COVID-19 worldwide [2, 7, 28]. Since the beginning of the pandemic, a lot of growing body of COVID-19 modeling studies estimating the COVID-19 morbidity and mortality burdens have been published (see, for instance, [5, 14, 29–34] and the references therein**)**.

Besides, small changes in the genetic code of viruses occur during transmission. These changes are called “mutations”. Most of the mutations are transient, and some may persist to become more severe outbreak. The genetically modified version of the virus with one or more new mutations from the original version is known as a “variant” [35]. According to the COVID-19 Genomic UK Consortium (COG-U.K.) [36], thousands of COVID-19 mutations are being detected, but few of them are likely to threaten public health [37–39]. Some studies shows that most viruses’ mutations are not harmful and could not cause any severe infection [38, 40]. The transmissibility and severity of SARS-CoV-2 likely increased due to some devastating mutations, such as the mutation in D614G amino acid. These evolved mutations may result from natural selection, and the steady increase of the G614 variant at regional stages could designate a fitness gain to this variant [41, 42]. This mutation could increase the efficiency of the viral cell fusion to the host cell. Therefore, these variants have higher transmission rates [43].

The P.1 variant with N501Y, E484K, and K417T mutations was first detected by the Japanese authorities on January 6, 2021, from four travellers who arrived at Tokyo Haneda Airport Japan after returning from Brazil four days earlier [44]. Health authorities and epidemiologists are still investigating if this variant is more severe, besides its higher transmission rate, or could detriment current diagnostics or vaccines [35, 38]. The P.1 lineage was linked with increased severity and reinfection scenarios [45–47]. Previous reports on SARS-CoV-2 genomic sequences highlighted that P.1 is more transmissible of up to 1.7–2.4 fold and previous infection by non-P.1 gives about 54–79% of the protection against P.1 infection compared with non-P.1 lineages [48]. The mutations of this variant include the N501Y, which has some similarities with the variants identified in South Africa and the United Kingdom (UK) [38]. Currently, three COVID-19 variants are considered the most dangerous ones, raising more public health concerns. These are the lineage B.1.1.7 variant identified in the UK with N501Y mutation (which has now evolved to include the E484K mutation in the UK) [49]; lineage B. 1.351 identified in South Africa; and the lineage P.1 variant identified in Brazil [36, 44]. These variants are called “variants of concern (VOCs)” [38, 50]. Since they can potentially reduce antibody neutralization and increase affinity for ACE2 receptors, which results in increased severity and could even lead to death, they are also linked to higher viral transmissibility, increased disease severity, and possible evasion of immunity, potentially impacting reinfection and vaccine effectiveness [9, 40, 51].

The P.1 variant has been detected in over 70 countries [44], including the United States, Canada, Belgium, Turkey, India, Brazil and Peru, as of September 29, 2021. It is currently storming across the South American region, leading to more severe cases and deaths even in places with high vaccination coverage. The resurgence of the second wave in South America would be an essential lesson for the rest of the world to tighten and improve the current control measures. Peru, a country of around 30 million people, is currently one of the world’s hardest-hit countries with a COVID-19 mortality of about 200,000 (which makes the death *per capita* the highest globally by June 1, 2021) [7, 52, 53]. A case resurgence was observed in April 2021 that has been declared the deadliest month for Peru since the pandemic began [52, 54, 55]. The P.1 variant was likely the cause of over 40% of the infections in Lima, the capital of Peru [55]. Health authorities are investigating another strain called C.37, which first emerged in Peru in August 2020 and has raised public health concerns, especially among the neighbouring countries such as Argentina, Chile, and Ecuador [56]. Colombia also experienced similar scenario with Peru, where occupancy in intensive-care units hit 90% in the capital, Bogotá, and hospitals in other cities were nearly overwhelmed [55]. Several cross-sectional studies suggested that the P.1 variant was up to 2.2 times more contagious and 61% more capable of reinfecting people than the original SARS-CoV-2 virus [38, 55, 57, 58]. Many countries in South America that experienced sharp increased in cases and deaths have, for the most part, not done extensive genomic sequencing to determine how many people been infected by P.1 [9]. Some reports show that the P.1 variant was the primary driver of the pandemic in the region [9]. Therefore, it is essential to determine infection fatality rate (IFR), infection attack rate (IAR) and reproduction number (${R}_{0}$), which helps shed more light and understanding on the transmission and control strategies of emerging and re-emerging infectious diseases, including the COVID-19.

The IFR, IAR and ${R}_{0}$ are some of the most crucial epidemiological parameters for estimating the actual burden of disease spreads and are used to assess the effectiveness of control measures. It is imperative to have accurate and up-to-date estimates of these parameters in different populations to develop essential benchmarks to understand the epidemics' spread to guide public health practitioners and policymakers in planning effective and sustainable policy for COVID-19 prevention and control. Furthermore, numerous epidemiological models have been proposed and used to study the transmission dynamics of SARS-CoV-2. For instance, a boosted regression tree (BRT) and multivariable logistic regression models were employed by Tao et al. [59] to identify the relative contribution and effect size of the risk factors associated with the asymptomatic cases and IFRs for COVID-19 in Hong Kong. They found that males and older cohorts were associated with higher IFR than females and younger cohorts. According to Buss et al., the basic reproduction number (${R}_{0}$) for Amazonas was estimated at 2.5–3.0 during the hugely unmitigated outbreak [10, 11], which indicates high transmission potential of the virus to spread and cause large outbreaks. Moreover, the expected infection attack rate (IAR, i.e., proportion of the total population being infected) in a homogeneously mixed population during unmitigated outbreak was estimated at 89–94% for Amazonas, Brazil [60]. When the percentage of infected individuals exceeds the herd immunity threshold of 60–67% (which can be calculated using the relation $100 \times (1-(1/{R}_{0})$), each infection generates less than one secondary case, thus, incidence declines [61].

This study investigates spatiotemporal variability of the COVID-19 pandemic, as well as estimates IAR, IFR and ${R}_{0}$ for the twelve most affected countries in South America, namely; Argentina, Bolivia, Brazil, Chile, Colombia, Ecuador, Guyana, Paraguay, Peru, Suriname, Uruguay, and Venezuela. Epidemiologically speaking, the higher number of these parameters indicates that the epidemic would persists and spreads in the population. We investigate the range of SARS-CoV-2 transmission scenarios in these countries and assess the pandemic's key drivers, such as pharmaceutical and NPIs measures. Notably, we examine the transmission trends and identify the main epidemic drivers in the region (e.g., human behavioral changes, social distancing, and minimal or partial compliance of other NPIs measures by the general public). Further, we will compare the results of each scenario to reveal possible reasons for the current fluctuations (rise and fall) in mortality that would help assess mitigation strategies and inform public health responses and policymakers for effective control of the outbreak.

## Methods

### Epidemic data

We retrieved the COVID-19 datasets from different sources. Epidemiological time-series datasets for COVID-19 cases and deaths are from publicly available reports collected and compiled by the WHO disease surveillance systems (dashboard) [2]. Similar data can also be obtained from Our World in Data via [62, 63]. In particular, confirmed cases and deaths in "Our World in Data" come from the COVID-19 Data Repository by the Center for Systems Science and Engineering (CSSE) at Johns Hopkins University (JHU) [64]. In case of vaccination, the data were obtained from [65]. Other datasets analyzed include (i) COVID-19 related data for Brazil, which was collected and compiled by the Brazil Ministry of Health disease surveillance report through the Secretariat Health Surveillance (SVS) available from [66, 67], (ii) state-level population data for Brazil [68], (iii) state-level datasets for COVID-19 cases and deaths for Brazil [69], (iv) state-level daily mortality datasets for Severe Acute Respiratory Illness (SARI) obtained from hospitalized patients (a good proxy of COVID-19 deaths) are available from [70], (v) SARG data (hospitalized SARI cases, first symptom onset date) for Brazil [71]; and (vi) additional information on the COVID-19 situation report for Peru were obtained from [72].

**Fig. 1:**
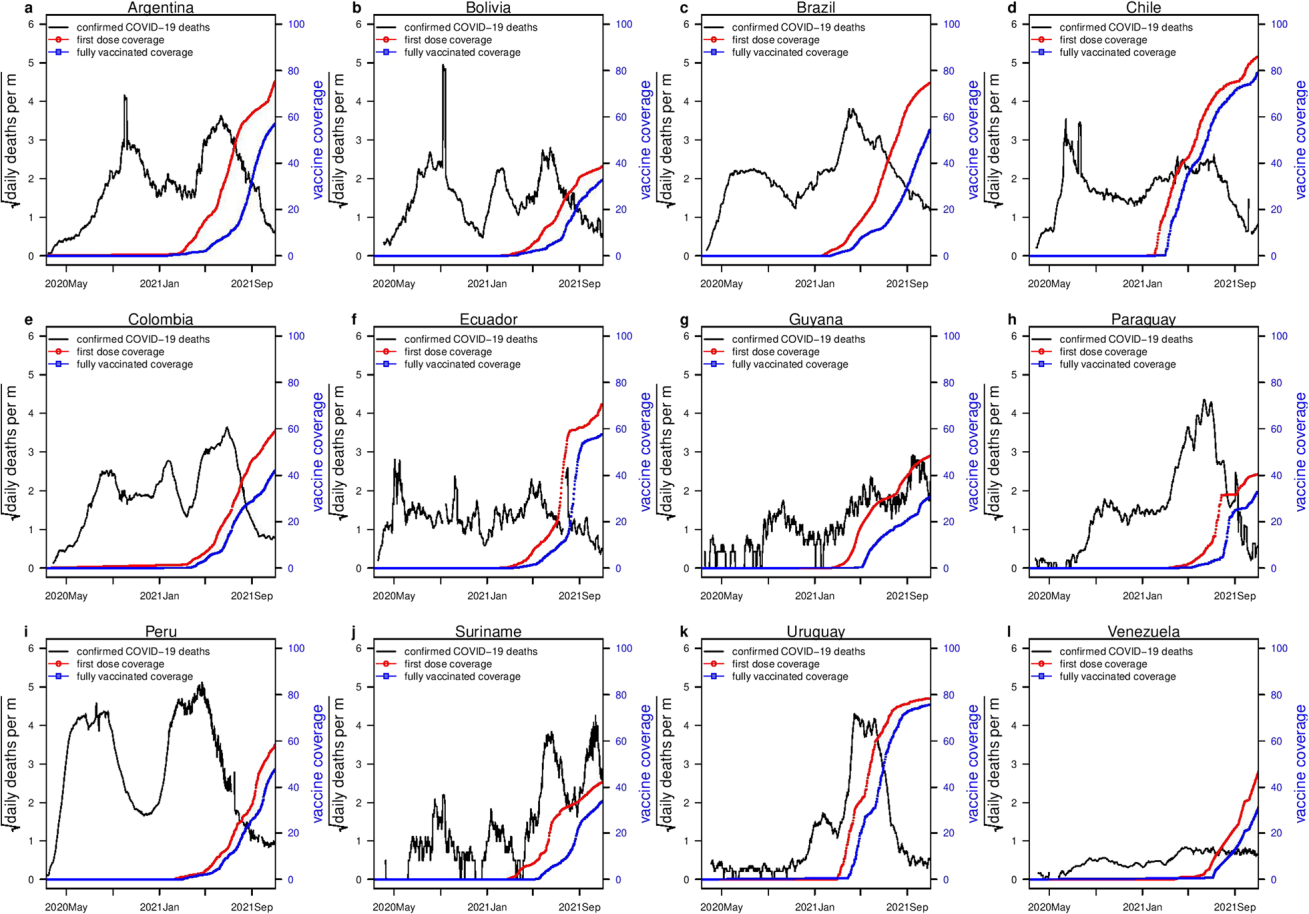
Simulations of the model with vaccination for the COVID-19 deaths per million population (black curve, in square root scale such that we can see the small values) for the 12 Southern American countries, and the red and blue curve, respectively, represent partly one-dose vaccinated and fully two-dose vaccinated individuals. The data were retrieved from Our World in Data accessible via [62, 65]. The figure was generated using the Free Statistical Software R with version 4.1.2

Our analysis focuses on the 12 most-affected countries with SARS-CoV-2 mortalities in South American region to estimate IFR, IAR and ${R}_{0}(t)$. For the geographical locations of these countries, see the map in Additional file 1: Fig. S3. For each country, the case incidence and mortality data were used. It is worth stating that the current study did not assess the individual patient’s data; hence no ethical approval and patient consent is required. Further, we used COVID-19 death for Peru after official correction [53]. Consequently, the revised death data in Peru is in line with "excess deaths" figures, which researchers have used in estimating the under-ascertainment of cases in Peru and other countries. Excess death accounts for the total number of extra deaths over time in comparison to the average level in the 5-year pre-pandemic period. For Brazil, we used the severe-acute-respiratory-infection-hospitalized deaths, larger than the official COVID-19 deaths cases and is believed to be more accurate reflection of the actual scenario. We show the reported COVID-19 deaths and vaccination coverage over time in Fig. 1.

### Epidemic model

**Fig. 2:**
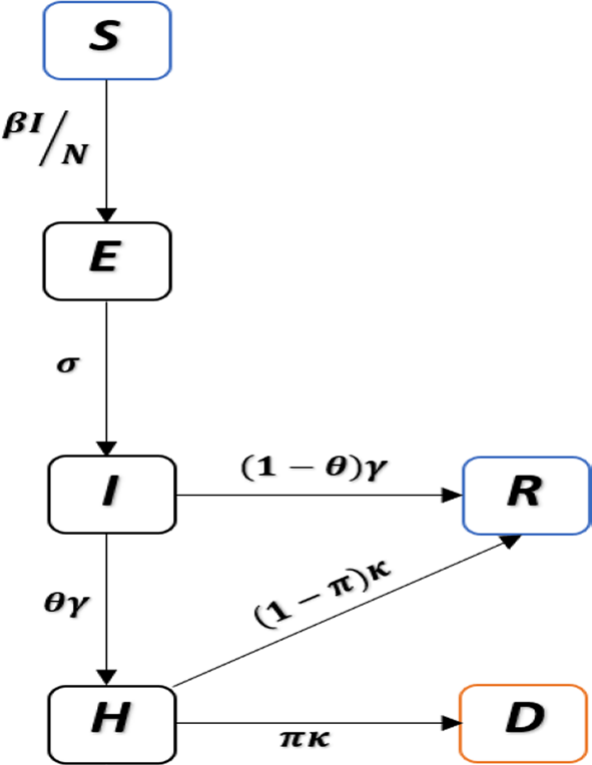
Schematic diagram of COVID-19 model without vaccination

We adopted a susceptible-exposed-infectious-recovered (SEIR)-based model with time-varying transmission rate $\beta (t)$ implemented as an exponential cubic spline function of time. Other key epidemic parameters were estimated to compare fitting performance in each scenario. We divided the total human population, $N(t)$, at time, $t$, into the mutually exclusive compartments of susceptible $S(t)$ (individuals who are at risk of the COVID-19 infection), exposed $E(t)$ (individuals who are exposed to COVID-19), infectious (including asymptomatic, mild, and severe cases) $I(t)$, hospitalized severe cases $H(t)$, and recovered $R(t)$ individuals. And the compartment $D(t)$ accounts for the total number of people who die due to COVID-19 infection. The model, represented in Fig. 2, is given by the following system of coupled differential equations. Details of the parameters of the model are given in Table 1.

**Table 1:** Parameters of the model

Parameter description	Symbol	Value
Time-varying transmission rate	$\beta (t)$	Variable
Infectiousness onset rate	$\sigma$	$1/2\,{\mathrm{ day}}^{-1}$
Rate of loss of infectiousness	$\gamma$	$1/3\,{\mathrm{ day}}^{-1}$
Removal rate of hospitalized cases	$\kappa$	$1/8\,{\mathrm{ day}}^{-1}$
Ratio of severe cases out of all infected cases	$\theta$	Variable
Proportion of mortality out of severe cases	$\pi$	Variable
Proportion of fully protected individuals due to vaccination	$\eta$	$0.85$
Proportion of susceptible individuals who received COVID-19 vaccine per day	$\tilde{v }(t)$	Based on data

$$\begin{array}{ll}\dot{S}=-\frac{\beta SI}{N}, \\ \dot{E}=\frac{\beta SI}{N}-\sigma E, \\ \begin{array}{ll}\dot{I}=\sigma E-\gamma I, \\ \dot{H}=\theta \gamma I-\kappa H, \\ \begin{array}{ll}\dot{D}=\pi \kappa H, \\ \dot{R}=\left(1-\theta \right)\gamma I+\left(1-\pi \right)\kappa H.\end{array}\end{array}\end{array}$$

In the above equations, the dot above the variables denotes the time differentiation. The parameter $\beta (t)$ represents the time-varying transmission rate, $\sigma$ is the infectiousness onset rate, $\gamma$ is the rate of loss of infectiousness, and $\kappa$ represents the removal rate (due to death or recovery) of hospitalized cases. The parameter $\theta$ denotes the ratio of severe cases out of all infected cases and $\pi$ represents the proportion of mortality out of severe cases. Hence, the overall CFR (or IFR) is equivalent to $\theta \pi$. We note that the exact definitions of $H$, $\theta$, and $\pi$ are not important, since we only fit death data, rather than infected or hospitalized cases. Thus, the most important parameter is the IFR, i.e., the product $\theta \pi$. In order to further simplify the model, we assume $\theta \approx \pi$, thus the IFR is ${\theta }^{2}$. Possible reason for making this assumption was due to the unavailability of COVID-19 hospitalized severe cases data. We have tested alternatively where one of the two is fixed at some small values and yielded similar results. The class of $H$ serve as an intermediate status (also a delay class) between infectives and deaths. We assumed all parameters of the model to be constant with the exception of time-varying $\beta (t)$. It is important to note that the demographic processes (i.e., births and natural deaths processes) are not included in the current model since the timescale of the COVID-19 pandemic is much shorter than the usual demographic timescale [73, 74].

Following previous studies [75, 76], we define the transmission rate, $\beta (t)$, as an exponential cubic spline function, i.e., $\beta (t)=\mathrm{exp}(cubic\_spline)$, with $n$ nodes evenly distributed over the study period. We set time step size to be one day and integrated $\dot{D}$ for one day and yielded the simulated daily deaths ${D}_{t}$. We defined the reported deaths as ${C}_{t}$, which follows a negative binomial distribution

$${C}_{t}\sim \mathrm{NegativeBinomial }\left(\mathrm{mean}={D}_{t}, \mathrm{variance}={D}_{t}\left({1+\tau D}_{t}\right)\right),$$

where $\tau$ denotes the over-dispersion of reporting, and accounts for the measurement noise due to surveillance and heterogeneity among individuals. When $\tau =0$, this is reduced to a Poisson distribution. Namely, we assumed the reporting is an over-dispersed Poisson process, which is widely used and biologically reasonable.

**Fig. 3:**
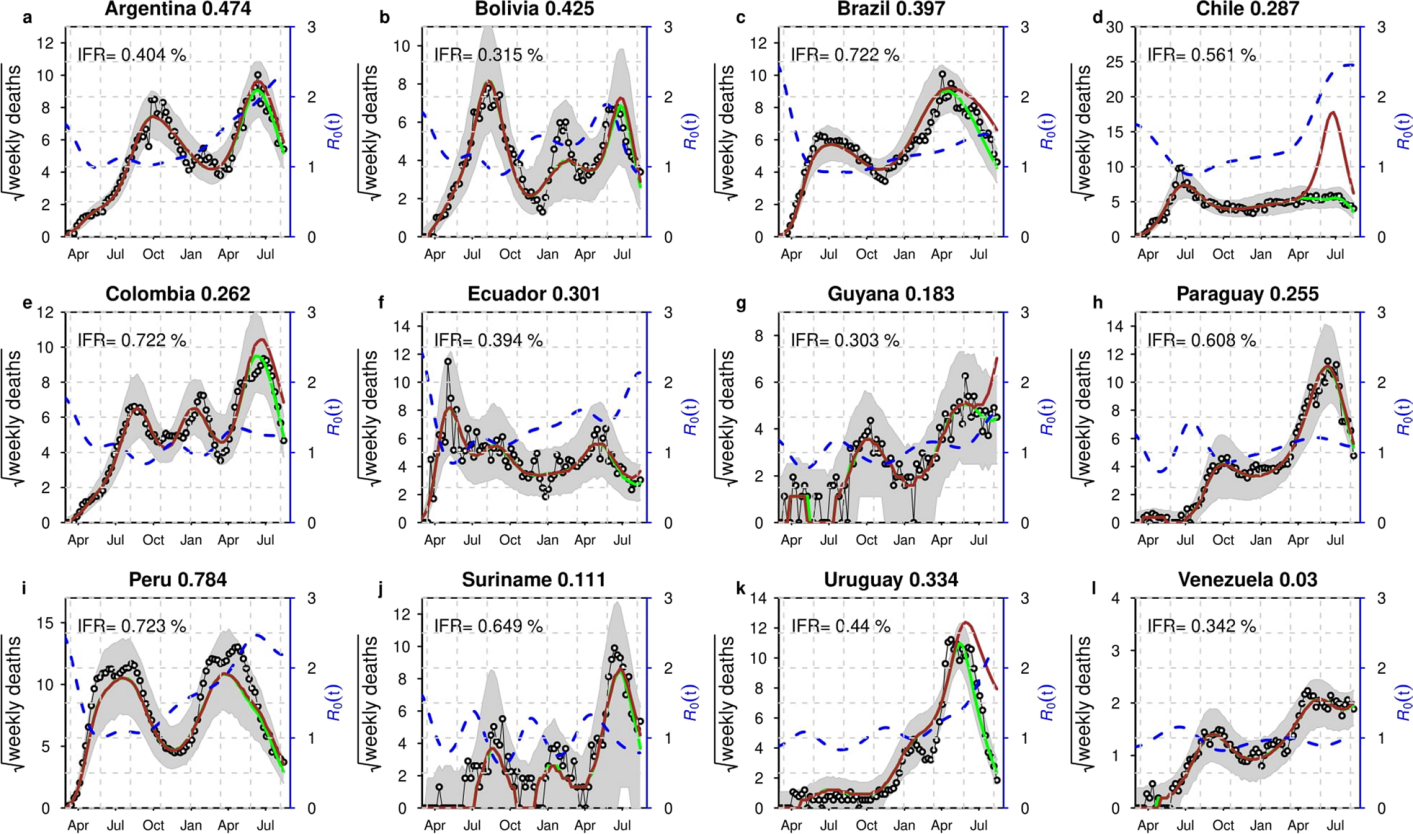
Model fitting results for the 12 South American countries with the highest COVID-19 deaths (represented by panels **a–l**). The time-series plots for the weekly reported COVID-19 deaths are represented in red line with simulation median (in black) and the basic reproduction number, ${R}_{0}(t)$, in the dashed blue line. The shaded region represents the 95% confidence interval of the simulation. The panels **a–l** represents the infection attack rate (IAR) for the 12 countries, respectively, with $\pi =\theta$, and $n=9$. The resurgence of deaths in Brazil and Peru could be explained by the resurgence of ${R}_{0}(t)$ due to the emergence of new variants and relaxing of nonpharmaceutical interventions measures. Note that $\pi , \theta$ and $n$ represent proportion of mortality out of severe cases, ratio of severe cases out of all infected cases, and number of nodes, respectively. The COVID-19 confirmed cases and deaths data come from [64]

Moreover, the following set of parameter values are used for the simulation results, that is, the mean latent period ($\sigma$) as two days, the mean infectious period ($\gamma$) as three days, and the mean duration of hospitalization ($\kappa$) as eight days. We assumed that due to the time discretization in the simulation, the period should be slightly be higher than these values. Thus, the sum of the mean latent period and infectious period are 6.07 days with a 1-day time step size (the sum approaches five days only when time step size approaches zero), which is close to the estimated generation time (GT, the sum of the mean latent and infectious period in an SEIR setting) by considerable amount of literature [14, 74, 77–80]. The mean duration from infection to death is about 14.57 days (with a 1-day time step size), which is biologically reasonable [81]. We note that many previous studies used longer generation time, which is against the reported GT estimates of COVID-19, which may lead to overestimating ${R}_{0}$ [74]. We assumed that the initial susceptibility proportion is 95% to reflect the fact that some proportion of the population (e.g., children) are less susceptible [82]. We assumed that the initial infectious population was lower than 10,000. The initial exposed and infectious populations are equal. The initial $H$ cases are 1/10 of the initial infectious cases. The initial deaths are 1/10 of those of the $H$ cases. We fixed $n=9$ in the cubic spline. We assumed $\theta$ between [0.055 and 0.085], thus IFR was between [0.3%, 0.72%], see Fig. 3. Similarly, the results in Additional file 1: Figs. S1, S2 for the transmission dynamics of COVID-19 in 12 South American countries obtained by varying $\theta$, fixed $\pi$, and different $n$. The estimates of IAR, IFR, and ${R}_{0}$ are roughly consistent. If we assume COVID-19 confirmed deaths are accurate, with estimated IFR, we can also estimate the total infection in each country and the IAR. Moreover, we used the standard approach of the next-generation matrix technique to compute ${R}_{0}$ (see Additional file 1: Sect. 6) [83], which determines the number of COVID-19 secondary cases generated by a typical case if an infected person is placed into an entirely susceptible population.

### Extended epidemic model with vaccination

Here, the initial model was extended by incorporating the vaccination scenario. The COVID-19 vaccination rate ($v(t)$) represents the proportion of a population administered with COVID-19 vaccine *per day*. The COVID-19 vaccination data can be retrieved via [2, 62, 65]. The rate at which susceptible individuals get vaccinated is given by $\tilde{v }(t)$= $v(t)/(1-{\int }_{0}^{t-}v(s)\mathrm{ds})$, where $\tilde{v }(t)$ represent the proportion of susceptible individuals who received a vaccine *per day*. We split the population into two subpopulations, i.e., fully vaccinated and not fully unvaccinated. We focus our analysis to the not fully unvaccinated group with the second dose or first dose for previously infected. The dynamic model is represented by the following coupled system of nonlinear ordinary differential equations.

$$\begin{array}{ll}\dot{S}=-\frac{\beta SI}{N}-\eta \tilde{v }S, \\ \dot{E}=\frac{\beta SI}{N}-\sigma E, \\ \begin{array}{ll}\dot{I}=\sigma E-\gamma I, \\ \dot{H}=\theta \gamma I-\kappa H, \\ \begin{array}{ll}\dot{D}=\pi \kappa H, \\ \dot{R}=\eta \tilde{v }S+\left(1-\theta \right)\gamma I+\left(1-\pi \right)\kappa H. \end{array}\end{array}\end{array}$$

The basic theoretical analysis of the model with vaccination was provided in Sect. S6. In the above model,  the parameter $\eta =0.85$ denotes the proportion of the population that becomes fully protected over the study period. The model did not consider reinfection scenario since it has been reported to be at low rate with considerably milder symptoms [8]. Considering that the infection risk is not uniformly homogenous and some individuals have stronger immunity than others, we assumed that at least 5% of the population have pre-existing cross-immunity from other coronaviruses [12]. The initial exposed and infectious people were equal and randomly chosen in the range [0, 0.001] from the total population. *H* class has 1/10 of that in the infectious class, and Death (D) class has 1/10 of that in the *H* class.

We adopted a partially observed Markov process (POMP) model using maximum likelihood-based iterated filtering technique to fit the mortality data [84]. One of the uniqueness of our proposed model is that it allows time-varying flexible transmission rate ($\beta (t)$), which was taken as an exponential cubic spline [85–87] to account for the simultaneous impact of the all-possible interventions, including vaccination. For details on the fitting processes, see pseudo code in Additional file 1: Sect. S7 and https://kingaa.github.io/sbied/.

## Results and discussion

In Fig. 3, we depicted the fitting results for the top twelve South American countries with COVID-19 mortality. The time series of weekly confirmed COVID-19 deaths was denoted as red line, the median of 1,000 simulations was denoted as black circle line, and the basic reproduction number is a blue dashed line, i.e., ${R}_{0}\left(t\right)=\beta (t)/\gamma$. The shaded region denotes the 95% confidence region of 1,000 simulations. We observed that there were disparities and similarities in the transmission rate across the twelve countries.

Figure 3a–l have similarities, as seen from the simulation results. Each country is experiencing (or have experienced) at least two waves of COVID-19 epidemic with different time-varying effective reproduction number (${R}_{0}\left(t\right)$). The first peak of COVID-19 deaths reached around mid-October to November 2020 in Argentina, Bolivia, Brazil, Colombia, Guyana, Paraguay, Suriname, and Venezuela. The first peak of COVID-19 deaths reached around July–August 2020, and the second peak reached around February 2021 in Chile, Ecuador, and Peru. By March–April 2021, the peak of the second wave in deaths was still in the process of descending in Argentina, Bolivia, Colombia, Suriname, and Uruguay. While, the peak of the second wave was still increasing in Brazil, Chile, Ecuador, Guyana, Paraguay, Peru, and Venezuela. The peak seems to be levelling out in Chile by around June–July, 2020. The trends of COVID-19 in Brazil and Peru, the hardest-hit countries (represented by the panel (c) and (i) of Fig. 3), have similarity. Based on the time-varying basic reproduction number, ${R}_{0}(t)$, each of these two countries show at least two-waves trends of COVID-19 mortalities, which is currently ongoing by September 2021. The peak of deaths slightly follows the peak of ${R}_{0}(t)$. The slightly ascending ${R}_{0}\left(t\right)$ in Brazil and Peru around December 2020 predicted a rise in death cases within the next couple of weeks. Further, we observed a slight difference in the reproduction number across the cities of Brazil and Peru (see Additional file 1: Figs. S3, S4), which could be due to the differences in human behaviour, NPIs compliance, and availability of health resources.

### Infection fatality rate

We estimated the COVID-19 IFR for the 12 South American countries with the most deaths, as shown inside each panel of Fig. 3. The estimated IFR (or infection to reported death ratio) is shown inside each panel (a) to (l). The IFR varies between 0.303% and 0.723%. Most countries in the region experienced similar trends of COVID-19 mortalities. Peru has been the hardest hit country and, thus, has the highest IFR of 0.723%. The IFR was approximately similar for Brazil, Chile, Colombia, Paraguay, and Suriname (with IFR of 0.772%, 0.516%, 0.722%, 0.608%, and 0.649%, respectively) and significantly higher than the IFR for other countries. Guyana has the lowest IFR, estimated at 0.303%, followed by Bolivia, Venezuela, Ecuador, Argentina, and Uruguay (with IFR of 0.315%, 0.342, 0.394, 0.404, and 0.44, respectively), probably due to underreporting of deaths [88]. We note that our IFR is smaller than the reported raw case-fatality rates since not all infections will be reported. For instance, according to Ramirez et al. [89], the raw case fatality rate of COVID-19 in Argentina, Bolivia, Chile, Paraguay, and Brazil, by June 3, 2021, was estimated at 3.11%, 3.42%, 1.09%, 1.09%, 5.61%, respectively [88]. Our estimated IFR for Brazil is lower than our estimated IFR in Manaus, Brazil [61], the most affected city in Brazil. Further, a recent report by Rivera et al. [90] revealed that the IFR for most countries in South America varies between 0.87% and 7.14%, with a 95% confidence interval of 0.34% to 10.76%.

### Infection attack rate

Similarly, we estimated the corresponding IAR for the twelve South American countries, see Fig. 3a–l. The IAR was shown in the title of each panel (a) to (l) (in bold font). The IAR varies between 0.03 and 0.784. By June 2021, Peru was the hardest-hit country with the highest COVID-19 mortality in terms of *per capita* rate. Peru has an estimated IAR of 78.4%, which means 78.4% of the population has been infected. The IAR for Peru was significantly higher than the rest of the South American region. Venezuela has the lowest IAR, which was estimated at 3%, likely due to better compliance of COVID-19 containment measures, such as better social distancing policies and other human behaviour factors. While for the remaining countries, the IARs vary between 11.1% and 47.4%. Most countries have also shown similar wave patterns in the region, especially as the second wave transmits faster in most countries.

### Assessing the effect of vaccination

We studied the model "with" and "without" vaccination to evaluate the effect of the vaccination on the overall transmission dynamics. The model without vaccination was reconstructed by rerunning the fitted model with $v(t)=0$. Our model was the simplest model to explore vaccination's effect. However, one could incorporate *V* as a compartment to represent the proportion of the vaccinated population with low immunity response and susceptible to breakthrough infection. Our preliminary testing/analysis showed that these two models yielded similar results (as in Fig. 3). Moreover, the proposed model captured well the epidemiological scenarios for COVID-19 dynamics in South America by identifying the key factors that enhanced the transmission in the region. For instance, social and environmental factors, human behaviors, partial or low compliance of pharmaceutical and NPIs measures, which in combination helps to investigate the actual dynamics of SARS-CoV-2 in South America and beyond. We also integrate our findings based on our proposed model to assess the duration and intensity of those factors, especially the pharmaceutical and NPIs measures that are crucial and needed to maintain or strengthen control and prevention of SARS-CoV-2.

In this paper, we chose one strain model rather than two strain model to examine the transmission patterns of SARS-CoV-2 in the South American countries since our death data are not strain-specific. We assumed full cross-immunity between strains, and most parameter settings are considered to be constant and identical to two strains (e.g., wild strain and P.1 strain). Although the two strains model could capture more dynamics and better explain the situation in the South American region, we used one strain model, which also works well with a flexible transmission rate. This work can be extended by employing two strain models to explore more dynamics features of SARS-CoV-2 in South America. Also, as future work, we plan to extend the model by adding more vital dynamics (e.g., demographic processes) to assess the model with and without vaccination qualitatively.

## Conclusions

In this study, we developed a methodology to estimate the IFR, IAR and reproduction number of COVID-19 in South American countries. In particular, we used an SEIR-typed model with time-varying flexible transmission rate. We observed that the initial reproductive number between countries in South America varies for some reasons, such as differences in vaccinations uptake, NPIs compliance, healthcare standards, and socioeconomic status. We also found reductions in IAR, IFR and reproductive number (transmission rate) corresponding to each wave, which were likely due to the differences in vaccination rates, human protective behavior reaction to recent deaths, or NPIs compliance. Those measures could be relaxed when the recent deaths decrease, and seems more biologically reasonable than assuming reproductive number to be constant. The drop-in reproductive number was not due to the depletion in susceptibility or transmissibility, since, in our model, we disentangled these two signals. Thus, this fluctuation in the transmission rate would lower the expected attack rate after each wave. Moreover, we also observed that the initial reproduction number of COVID-19 for hardest-hit countries (such as Brazil and Peru) was high, owing to the low NPIs compliance and fewer vaccines uptake. Overall, our results suggested that increasing the vaccination rate coupled with NPIs and other basic preventive measures, such as providing adequate medical resources and improving public health awareness programmes, could effectively suppress the pandemic’s impact in South America.

## Supplementary Information

**Additional file 1.** Supplementary figures and tables.

## Data Availability

All the data used can be found in the public domain, available from https://covid19.who.int/.
