# Supplementary material for: Infection fatality rate and infection attack rate of COVID-19 in South American countries
Source: Infect Dis Poverty. 2022 Apr 6;11:40. doi: 10.1186/s40249-022-00961-5 (PMC8983329; doi:10.1186/s40249-022-00961-5)
Supplement: Supplementary file 1 — Additional file 1. Supplementary figures and tables. [file 40249_2022_961_MOESM1_ESM.docx]

**Full Title**: Infection fatality rate and infection attack rate of COVID-19 in South American countries

**Running Title**: Transmission dynamics of SARS-CoV-2 in South America

Salihu Sabiu Musa^1,2^, Amna Tariq^3^, Liu Yuan^1^, Wei Haozhen^1^, & Daihai He^1, #^

1 Department of Applied Mathematics, Hong Kong Polytechnic University, Hong Kong, China

2 Department of Mathematics, Kano University of Science and Technology, Wudil, Nigeria

3 Department of Population Health Sciences, School of Public Health, Georgia State University, Atlanta, GA, USA

# Corresponding Author: [daihai.he@polyu.edu.hk](mailto:daihai.he@polyu.edu.hk).

**Supplementary Material**

**Contents**

1. Section 1: Fitting SEIHRD model to daily reported deaths for COVID-19 in 12 South American countries with most deaths
2. Section 2: Fitting SEIHRD model to daily reported deaths for COVID-19 in 27 states of Brazil
3. Section 3: Fitting SEIHRD to daily reported deaths for COVID-19 in 25 states of Peru
4. Section 4: Geographical location of the 12 South American countries
5. Section 5: COVID-19 vaccination scenario in South American countries
6. Section 6: Basic theoretical analysis of the model with vaccination
7. The pseudo-code is: 
   (1) prepare model equations, initial guess of parameter values, and data. 
   (2) use pfilter of POMP package to calculate Likelihood of model given data 
   (3) use mif function of POMP package to obtain the maximum likelihood estimate of parameters 
   (4) Repeat 2) and 3) until the maximum log likelihood is converged. 
   (5) Use simulate function of POMP package to obtain the model simulation 
   For step by step explanation and hand-on examples on the methodology, see https://kingaa.github.io/sbied/.

**Section 1:** Fitting SEIHRD model to daily reported deaths for COVID-19 in 12 South American countries with most deaths

In **Figure S1**, we fitted the SEIHRD model for 12 South American countries with highest reported COVID-19 deaths using similar approach as in the main text with $\pi=\theta$.


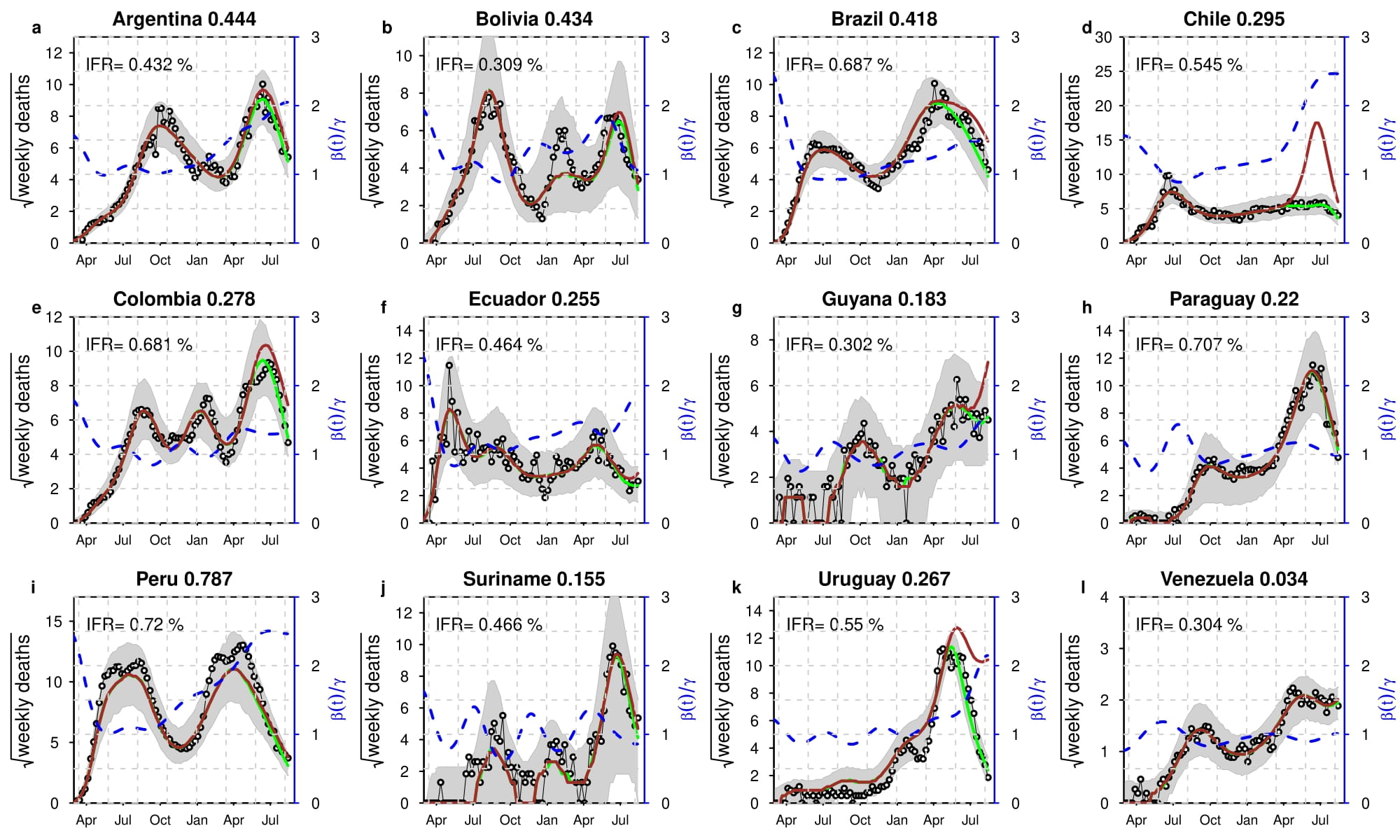


**Figure S1:** Fitting results for the 12 South American countries with highest reported COVID-19 deaths (represented by panels a-ls). Time series plots for the weekly reported COVID-19 deaths (in red circle) with simulation median (in black) and the basic reproduction number, $R_{0}(t)$, in dashed blue line. The shaded region represents the 95% confidence interval of the simulation. The numbers from each panels **a**-**l** represent the infection attack rate (IAR) for the 12 countries, respectively. With $\pi=0.072$ fixed, $\theta[0.042, 0.1],$ $n=9,$and infection fatality rate (IFR) between 0.3% - 0.72%.

In **Figure S2**, we fitted the SEIHRD model for 12 South American countries with highest reported COVID-19 deaths using similar approach as in the main text, with varying $\pi$ and $\theta$.


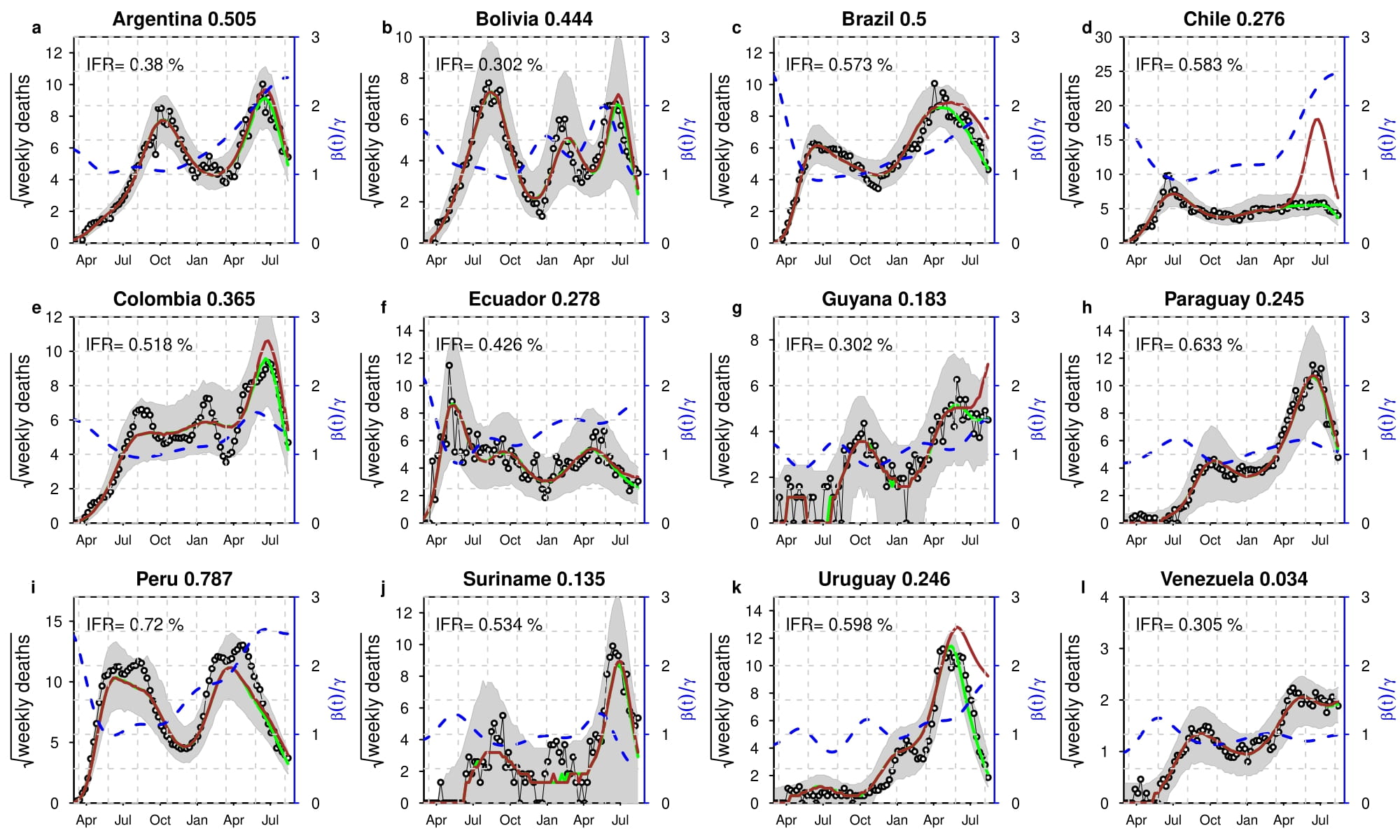


**Figure S2:** Fitting results for the 12 South American countries with highest reported COVID-19 deaths (represented by panels a-ls). Time series plots for the weekly reported COVID-19 deaths (in red circle) with simulation median (in black) and the basic reproduction number, $R_{0}(t)$, in dashed blue line. The shaded region represents the 95% confidence interval of the simulation. The numbers from each panels **a**-**o** represent the infection attack rate (IAR) for the 12 countries, respectively. With $\pi=0.072$ fixed, $\theta[0.042, 0.1],$ $n=8,$and infection fatality rate (IFR) between 0.3% - 0.72%.

**Section 2:** Fitting SEIHRD model to daily reported deaths for COVID-19 in 27 states of Brazil

In **Figure S3**, we fitted the SEIHRD model to daily death in 27 states of Brazil using similar approach as in the main text.

**Figure S3.** Fitting SEIRD model to daily reported death in 27 states of Brazil. The time series plots for the weekly reported COVID-19 deaths (in red circle) with simulation median (in black) and the time-varying effective reproduction number, $R_{0}(t)$, in dashed blue line. The shaded region represents the 95% confidence interval of the simulation. Each panel represent a state in Brazil (more details for 27 Brazilian states can be found via https://en.wikipedia.org/wiki/List_of_Brazilian_states_by_population).

**Section 3:** Fitting SEIHRD to daily reported deaths for COVID-19 in 25 states of Peru

In this case, we presented reasonable fitting results for the SEIHRD model to daily reported deaths for COVID-19 in all the 25 states of Peru. We estimated the time-varying effective reproduction umber in each case as given in **Figure S2** below.


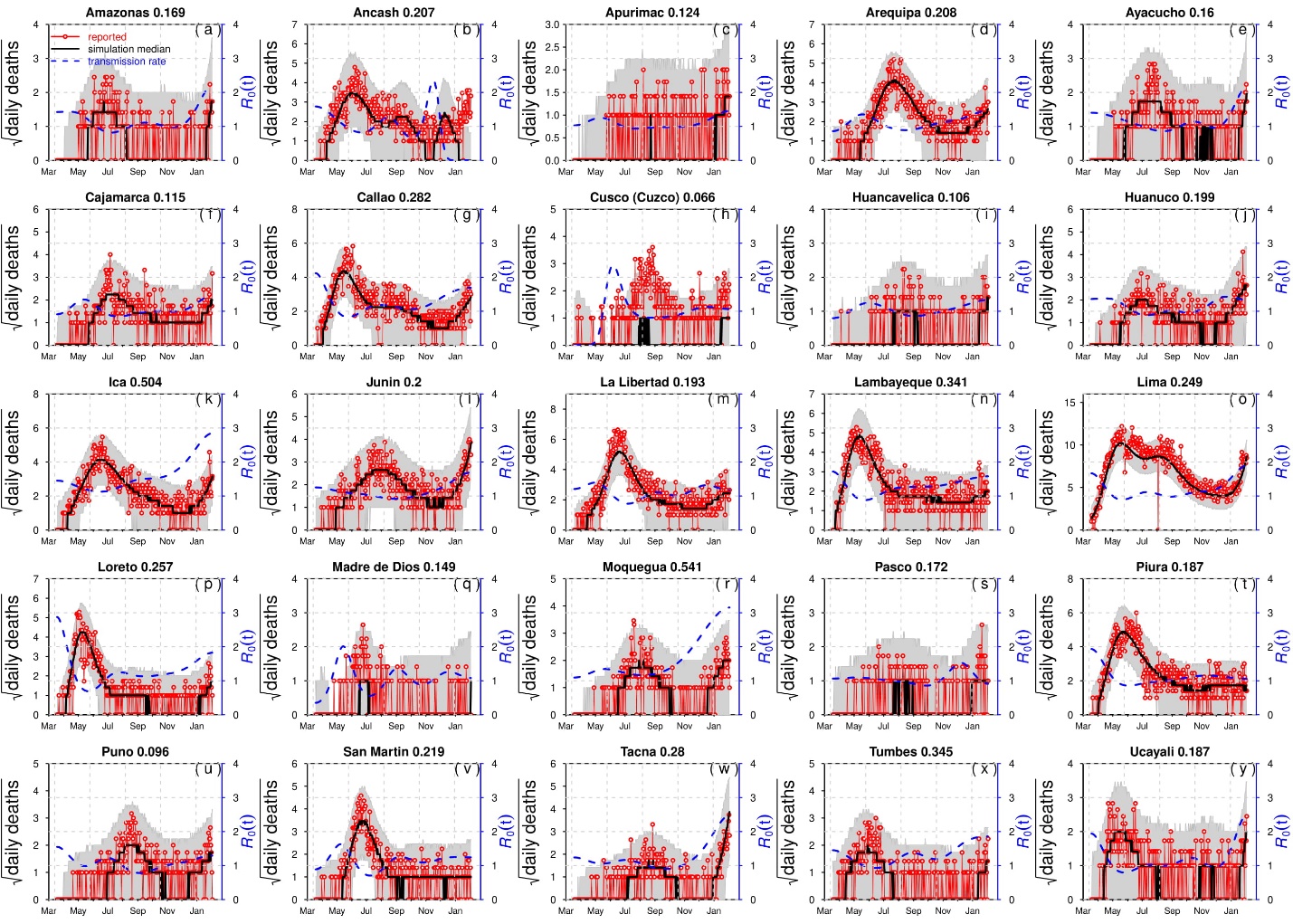


**Figure S4.** Fitting results of SEIHRD model to daily reported deaths for COVID-19 in 25 Peru states.

**Section 4**: Geographical location of the 12 South American countries

In this case, we depicted the twelve South American countries considered in the current study.

[1]
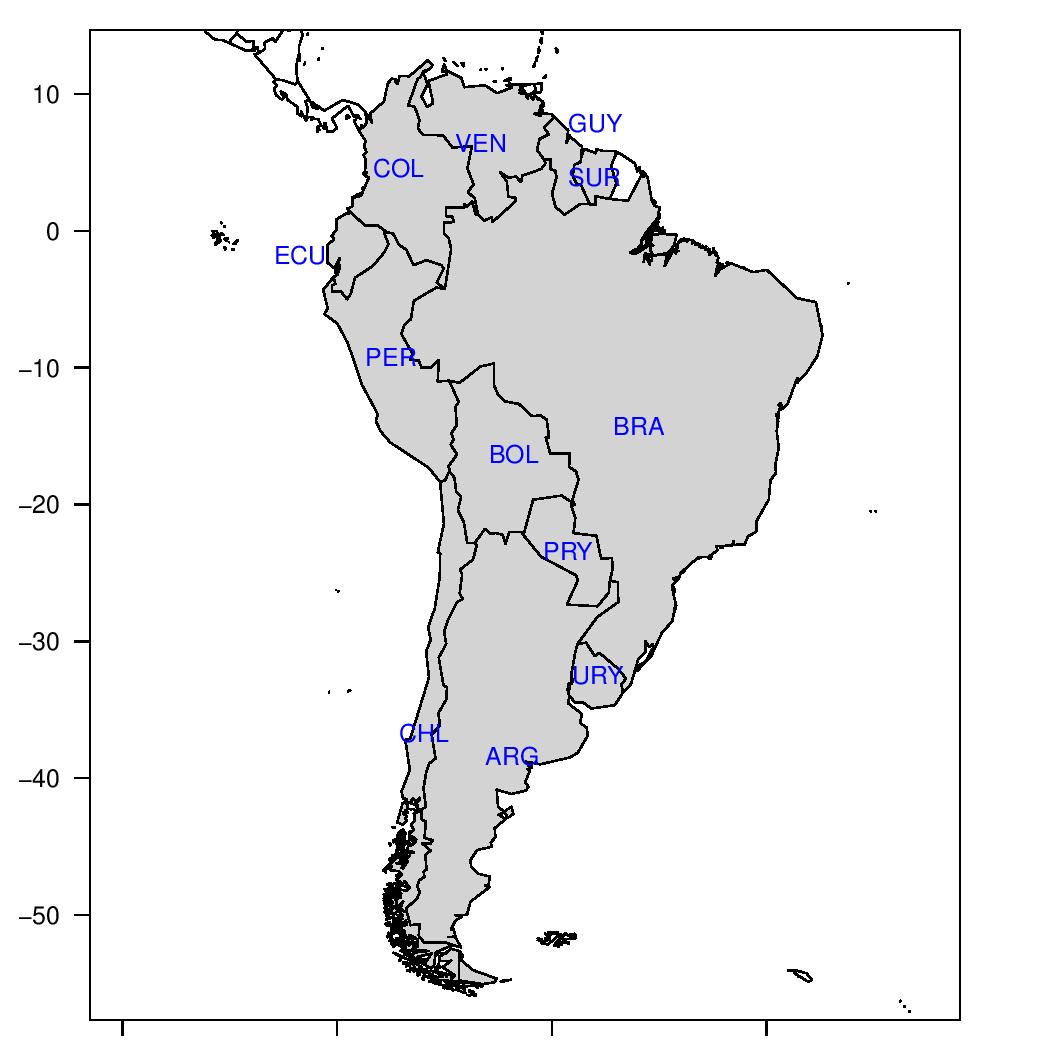


**Figure S5.** Geographical location of the 12 South American countries. The abbreviation ARG, BOL, BRA, CHL, COL, ECU, GUY, PRY, PER, SUR, URY, VEN, represent Argentina, Bolivia, Brazil, Chile, Columbia, Ecuador, Guyana, Paraguay, Peru, Suriname, Uruguay, Venezuela, respectively. We draw the map with the R package Shapefiles [2] and boundary data from [1].

**Section 5:** COVID-19 vaccination scenario in South American countries

**Table S1**: The COVID-19 vaccination situation in South America by October 4, 2021 [3, 4].

| Country | Vaccine doses (million) | Vaccine type (mostly used) | % of population fully vaccinated | % of population partly vaccinated |
| --- | --- | --- | --- | --- |
| Argentina | 52,083,872 | Sputnik V | 50.49 | 15.11 |
| Bolivia | 6,700,654 | Sputnik V | 27.54 | 8.40 |
| Brazil | 225,795,545 | Coronavac | 44.27 | 27.04 |
| Chile | 32,114,960 | Coronavac | 73.78 | 7.56 |
| Colombia | 40,404,815 | Coronavac | 33.56 | 18.83 |
| Ecuador | 20,841,350 | Pfizer/BioNTech | 55.45 | 8.00 |
| Guyana | 543,180 | NA | 23.53 | 21.67 |
| Paraguay | 4,640,590 | NA | 25.95 | 12.37 |
| Peru | 26,543,168 | Coronavac | 34.44 | 14.07 |
| Suriname | 401,242 | NA | 29.66 | 9.67 |
| Uruguay | 6,264,161 | Pfizer/Sinovac | 74.19 | 4.38 |
| Venezuela | 15,714,857 | Sputnik V/ Coronavac | 20.84 | 13.06 |

**Section 6:** Basic theoretical analysis of the model with vaccination

The basic reproduction number ($R_{0}$) is computed using the next generation operator approach [5] for the special case when $\beta\left( t \right)=\beta$, so that $R_{0}=\beta(t)/\gamma$. It is convenient, first of all, to define the following feasible region for the model with vaccination given by $\Omega=\left\{ \left( S,E,I,H,D,R \right)\in\mathbb{R}_{+}^{6} :S+E+I+H+D+R \leq N^{0} \right\}$. The region $\Omega$ is positively-invariant and attracting. Thus, we determine the equilibrium points by setting the right-hand side of the model with vaccination to zero as follows:

$$\begin{matrix} -\frac{\beta SI}{N}-\eta\tilde{v}S=0, \\ \frac{\beta SI}{N}-\sigma E=0, \\ \begin{matrix} \sigma E-\gamma I=0, \\ \theta\gamma I-\kappa H=0, \\ \begin{matrix} \pi\kappa H=0, \\ \eta\tilde{v}S+\left( 1-\theta\right)\gamma I+\left( 1-\pi\right)\kappa H=0. \end{matrix} \end{matrix} \end{matrix}$$

When there is no COVID-19 disease in the population, the *disease-free* equilibrium point (DFE) exists, denoted by $E^{0}$ and is given by $E^{0}=\left( S^{0},E^{0},I^{0},H^{0},D^{0},R^{0} \right)=\left( S^{0},0,0,0,0,0 \right)$.

Following previous studies (11,21), the result below is claimed.

Theorem 1: The DFE ($E^{0}$) of the model with vaccination is *locally-asymptotically* stable (LAS) if $R_{0}<1$, and unstable if $R_{0}>1$ (i.e., the epidemic grows, reaches a peak, and then decreases to zero as $t\to\infty$).

Theorem 2: The DFE ($E^{0}$) is *globally-asymptotically* stable (GAS) in the region of attraction $\Omega$ whenever $R_{0}\leq1$.

For the proof of the above theorems, see theorem 2 of Vanden Driessche & Watmough [5] and LaSalle’s Invariance Principle in [6], respectively. Epidemiologically, the consequences of the above theorems are interpreted respectively as: (i) A small inflow of COVID-19 cases will not generate an outbreak if the reproduction number is less than unity. (ii) The COVID-19 eradication depends on the initial size of the sub-populations of the proposed model, i.e., the initial number of infected humans introduced into the community does not have to be within the basin of attraction DFE, $E^{0}$, of the model.

**Section 7:** The pseudo-code is: 
(1) prepare model equations, initial guess of parameter values, and data. 
(2) use pfilter of POMP package to calculate Likelihood of model given data 
(3) use mif function of POMP package to obtain the maximum likelihood estimate of parameters 
(4) Repeat 2) and 3) until the maximum log likelihood is converged. 
(5) Use simulate function of POMP package to obtain the model simulation 
For step by step explanation and hand-on examples on the methodology, see https://kingaa.github.io/sbied/.

**References**

1. STAT Silk. <https://www.statsilk.com/maps/download-free-shapefile-maps>. Accessed 10 Nov 2021.

2. Shapefiles: Read and Write ESRI Shapefiles. https://cran.r-project.org/web/packages/shapefiles/index.html. Accessed 10 November 2021.

3. World Health Organization. Coronavirus disease (‎COVID-19)‎ Dashboard. <https://covid19.who.int/>. Accessed 10 Oct 2021.

4. Our World in Data. Coronavirus (‎COVID-19)‎ Vaccinations. <https://ourworldindata.org/covid-vaccinations>. Accessed 6 October 2021.

5. Van den Driessche P, Watmough J. Reproduction numbers and sub-threshold endemic equilibria for compartmental models of disease transmission. Math Biosci. 2002;180(1-2):29-48.

6. La Salle JP. The stability of dynamical systems: SIAM. 1976.
